# Experimental chronic kidney disease attenuates ischemia-reperfusion injury in an *ex vivo* rat lung model

**DOI:** 10.1371/journal.pone.0171736

**Published:** 2017-03-14

**Authors:** Chung-Kan Peng, Kun-Lun Huang, Chou-Chin Lan, Yu-Juei Hsu, Geng-Chin Wu, Chia-Hui Peng, Chin-Pyng Wu, Khee-Siang Chan

**Affiliations:** 1 Division of Pulmonary and Critical Care, Department of Internal Medicine, Tri-Service General Hospital, National Defense Medical Center, Taipei, Taiwan, Republic of China; 2 Institute of Undersea and Hyperbaric Medicine, National Defense Medical Center, Taipei, Taiwan, Republic of China; 3 Division of Pulmonary Medicine, Taipei Tzu Chi Hospital, Buddhist Tzu Chi Medical Foundation, Taipei, Taiwan, Republic of China; 4 School of Medicine, Tzu Chi University, Hualien, Taiwan, Republic of China; 5 Division of Nephrology, Department of Medicine, Tri-Service General Hospital, National Defense Medical Center, Taipei, Taiwan, Republic of China; 6 Division of Pulmonary Medicine, Department of Internal Medicine, Taoyuan Armed Forces General Hospital, Lungtan, Taoyuan, Taiwan, Republic of China; 7 Division of Clinical Nutrition, Taoyuan Armed Forces General Hospital, Lungtan, Taoyuan, Taiwan, Republic of China; 8 Department of Critical Care Medicine, Li-Shin Hospital, Taoyuan, Taiwan, Republic of China; 9 Department of Critical Care Medicine, Chi-Mei Medical Center, Tainan, Taiwan Republic of China; National Institutes of Health, UNITED STATES

## Abstract

Lung ischemia reperfusion injury (LIRI) is one of important complications following lung transplant and cardiopulmonary bypass. Although patients on hemodialysis are still excluded as lung transplant donors because of the possible effects of renal failure on the lungs, increased organ demand has led us to evaluate the influence of chronic kidney disease (CKD) on LIRI. A CKD model was induced by feeding Sprague-Dawley rats an adenine-rich (0.75%) diet for 2, 4 and 6 weeks, and an isolated rat lung *in situ* model was used to evaluate ischemia reperfusion (IR)-induced acute lung injury. The clinicopathological parameters of LIRI, including pulmonary edema, lipid peroxidation, histopathological changes, immunohistochemistry changes, chemokine CXCL1, inducible nitric oxide synthase (iNOS), proinflammatory and anti-inflammatory cytokines, heat shock protein expression, and nuclear factor-κB (NF-κB) activation were determined. Our results indicated that adenine-fed rats developed CKD as characterized by increased blood urea nitrogen and creatinine levels and the deposition of crystals in the renal tubules and interstitium. IR induced a significant increase in the pulmonary arterial pressure, lung edema, lung injury scores, the expression of CXCL1 mRNA, iNOS level, and protein concentration of the bronchial alveolar lavage fluid (BALF). The tumor necrosis factor-α levels in the BALF and perfusate; the interleukin-10 level in the perfusate; and the malondialdehyde levels in the lung tissue and perfusate were also significantly increased by LIRI. Counterintuitively, adenine-induced CKD significantly attenuated the severity of lung injury induced by IR. CKD rats exhibited increased heat shock protein 70 expression and decreased activation of NF-κB signaling. In conclusion, adenine-induced CKD attenuated LIRI by inhibiting the NF-κB pathway.

## Introduction

Lung ischemia reperfusion injury (LIRI) is a common form of lung injury caused by the reintroduction of blood flow or oxygen to the ischemic lung parenchyma. Clinically, LIRI is frequently encountered in patients undergoing lung transplantation who have experienced prolonged ischemia due to a complete cessation of blood flow and ventilation [1]. Even with procedural advancements such as cardiopulmonary bypass, donor-recipient risk stratification, lung preservation, pharmacological immunosuppression, and perioperative care, approximately 15% to 40% of the patients who undergo lung transplantation experience graft complications due to LIRI [2,3].

LIRI is typically characterized by increased microvascular permeability, increased pulmonary vascular resistance, pulmonary hypertension, diffuse alveolar damage, lung edema, and hypoxemia [2]. Experimental studies have demonstrated that the generation of reactive oxygen species, activation of the immune and coagulation systems, endothelial dysfunction, and apoptotic cell death may be molecular mechanisms underlying LIRI [2,4,5,6]. Despite the fact that neutrophil reduction, antioxidant supplementation, the use of free radical scavengers and the administration of vasodilators have been shown to attenuate LIRI *in vivo* [7,8,9,10,11], patients undergoing lung transplantation who experience LIRI still have an in-hospital mortality rate of 40% [12]. Thus, optimal allograft selection and donor pool expansion are crucial for improving lung transplantation outcomes.

Chronic kidney disease (CKD) has emerged as a global health issue, although advances in dialysis care and renal transplantation have significantly improved outcomes for patients with end-stage renal disease (ESRD). Respiratory complications are frequently encountered in patients with CKD, including reduced carbon monoxide diffusion capacity, pulmonary edema, uremic pneumonitis, fibrinous pleuritis, metastatic pulmonary calcification, and bronchopulmonary infections [13]. Due to concerns about the quality of the donor allograft, patients with CKD or ESRD on dialysis therapy are not generally considered acceptable lung transplant donors. However, some studies have reported only a minor and clinically insignificant reduction of the pulmonary function in ESRD patients on maintenance dialysis [14,15]. Moreover, successful transplants from dialysis-dependent donors have been reported[16]. Because lung donor shortage is still a major limiting factor for lung transplantation, it is critical to elucidate whether CKD patients are suitable as organ donors.

The molecular and cellular mechanisms that underlie the link between the kidneys and lungs have not been thoroughly studied. However, emerging evidence suggests the existence of cross-talk between the kidneys and lungs. Recent studies have demonstrated that lung function is negatively affected by increased alveolar proinflammatory cytokines, chemokines, macrophage-mediated pulmonary vascular permeability and caspase-dependent apoptosis, as well as decreased epithelial sodium channel and aquaporin-5 expression in experimental models of ischemic acute kidney injury[17]. Experimental studies exploring the molecular impact of CKD on lung injury are still lacking despite the clinical relevance of CKD in patients with respiratory manifestations.

The aim of the present study was to investigate whether CKD modulates the course of LIRI and its underlying molecular mechanisms. Our results demonstrate that experimental CKD could ameliorate LIRI by inhibiting nuclear factor-κB (NF-κB) signaling, a crucial signaling pathway involved in LIRI.

## Materials and methods

### Isolated perfused lung preparation

The animal care and experimental protocol were approved by the National Science Council and Animal Review Committee at the National Defense Medical Center in Taipei, Taiwan. The isolated-perfused *in situ* rat lungs were prepared as described previously[18]. Briefly, male Sprague Dawley (SD) rats weighing 300–350 g were anesthetized intraperitoneally with a 2:1 mixture of Zoletil and Rompun (1–1.5 ml/kg). Tracheostomy was performed to enable ventilation with a rodent ventilator (Model 7025, Ugo Basile, Varese, Italy). The lungs were ventilated with humidified air containing 5% CO_2_ at a frequency of 60 cycles/min, a tidal volume of 3 ml, and an end-expiratory pressure of 1 cm H_2_O. After a median sternotomy was performed, heparin (1 U/g of body weight) was injected into the right ventricle, from which 10 mL of blood was collected. This blood sample was mixed with 10 mL of physiological saline containing 2% bovine albumin, 119 mM NaCl, 4.7 mM KCl, 1.17 mM MgSO_4_, 22.6 mM NaHCO_3_, 1.18 mM KH_2_PO_4_, 1.6 mM CaCl_2_, 5.5 mM glucose, and 50 mM sucrose. Bovine albumin was added at a concentration of 4 g/dl to maintain the osmolarity of the perfusate. A wide-bore cannula was inserted into the left atrium via the left ventricle to collect the effluent perfusate for recirculation. The 10 ml of collected blood was added to the perfusate before initiating recirculation with the “half-blood” solution. The perfusion rate of the roller pump was set at 7ml/min and constant temperature (37°C) was maintained by a water bath. The isolated-perfused lungs remained *in situ* and the whole rat was placed on an electronic balance. Both the pulmonary arterial pressure (PAP) and the pulmonary venous pressure (PVP) were recorded from side arms of the inflow and outflow cannulae.

### Induction of lung ischemia and reperfusion

After the model was established, the lungs were subjected to 30 min of ischemia by arresting the ventilation and perfusion. After this period of ischemia, the lungs were reperfused and ventilated for 90 min.

### Determination of the microvascular permeability

An index of the microvascular permeability to water (filtration coefficient, *K*_f_) was determined from the lung weight change induced by an elevation in venous pressure. During ventilation and lung perfusion, the PVP was rapidly elevated by 10 cm H_2_O for at least 7 min. The slow, steady weight gain as a function of time (ΔW/ΔT) was plotted in a semi-logarithmic manner. The slow component then was extrapolated to zero time to obtain the initial rate of transcapillary filtration. From this plot, *K*_f_ was defined as the y-intercept divided by PVP (10 cmH_2_O) and lung weight, and it was expressed in whole units of g^**.**^min^-1.^cmH_2_O^-1^ × 100 g [19].

### Measurement of the lung weight/body weight and wet/dry weight ratios

After the experiments, the lungs were removed and massed to calculate the lung weight/body weight (LW/BW) ratio. A section of the right middle lobe of the lung was placed in an oven at 60°C for 48 h to determine the wet/dry (W/D) lung weight ratio.

### Protein concentration in the bronchoalveolar lavage fluid

Bronchoalveolar lavage fluid (BALF) was obtained at the end of the experiment by irrigating the left lung twice with 2.5 ml of saline. This fluid was centrifuged at 200×g for 10 min, and the protein concentration in the supernatant was determined using the bicinchoninic acid (BCA) protein assay reagent kit (Pierce, Rockford, IL, USA).

### Determination of the malondialdehyde level in lung tissue

The extent of lung tissue lipid peroxidation was determined by measuring the concentration of thiobarbituric acid-reactive substances using a commercially available enzyme-linked immunosorbent assay (ELISA) kit (Cayman Chemical Co., Ann Arbor, MI, USA) according to the manufacturer’s instructions. Briefly, the lung tissue was homogenized in radioimmunoprecipitation assay buffer with protease inhibitors. Malondialdehyde (MDA) solutions (0–50 μM) were used to generate a standard curve. Standards or samples were incubated with sodium dodecyl sulfate solution and a thiobarbituric acid solution containing thiobarbituric acid and trichloroacetic acid at 100°C for 60 min. The reaction mixtures were then centrifuged at 16,000×g for 20 min. The supernatants were removed and the absorbance at 532 nm was measured. The thiobarbituric acid-reactive substance values are expressed as malondialdehyde equivalents (nmol malondialdehyde/mg protein).

### MPO and CXCL1 immunohistochemistry (IHC) staining

Formalin-fixed paraffin sections (4-μm) were deparaffinized before antigen retrieval and endogenous peroxidase was blocked using 3% H_2_O_2_ in methanol for 15 min. The slides were then incubated for 60 min with a MPO rabbit polyclonal antibody (1: 800 dilution; Thermo Scientific) and a CXCL1 rabbit polyclonal antibody (1: 100 dilution; Bioss Antibodies). After washing, slides were sequentially incubated with rat tissue specific horseradish peroxidase-polymer anti-rabbit antibody (Nichirei Corporation) for 30 min. The horseradish peroxidase was then reacted with DAB substrate for 3–5 min, and the sections were then counterstained with hematoxylin. Images were captured using a fluorescence microscope (Leica / DM 2500) equipped with an EMCCD camera, using SPOT 4.7 advanced imaging software.

### Histopathological analysis

The right upper lung lobe was stained with hematoxylin and eosin. A histopathological assessment of the stained samples was performed by two pathologists blinded to the experimental conditions. For each section, 10 random areas were examined at a magnification of ×400. Within each field, the lung injury was scored according to (1) the infiltration or aggregation of neutrophils in the airspace or vessel wall, and (2) the thickness of the alveolar wall. Each assessment was graded 0, 1, 2, or 3, for no, mild, moderate, or severe injury, respectively. The resulting scores were added and presented as the lung injury score [20].

### mRNA extraction and Reverse Transcriptase(RT)-Polymerase Chain Reaction (PCR) of CXCL1

Total RNA was extracted from homogenized lung tissue using Direct‐zol™ RNA MiniPrep kit (ZYMO, USA). Single-strand cDNA was synthesized using MMLV Reverse Transcription Kit (Protech, Taiwan). Real-time PCR and subsequent calculations were performed with LineGene 9660 (Bioer, Germany). Quantitative analysis of gene expression associated with lung tissue were performed using rat-specific TaqMan® Gene Expression assays for CXCL1 based on expression of the reference gene (beta -actin). The primer sets were as follows: rat CXCL1; Assay ID Rn00578225_m1, and for rat beta -actin; Assay ID Rn00667869_m1. Real-time PCR was performed with Smart quant probe 2X master mix reagents (TIB MOLBIOL, Germany).

### Measurement of the concentrations of inflammatory mediators

The level of tumor necrosis factor-alpha (TNF-α) in the BALF, the TNF-α, interleukin-10 (IL-10) and MDA levels in the perfusate, and the heat shock protein 70 (HSP70) levels of lung tissue homogenates were measured after the experiment. The levels of TNF-α and IL-10 were determined using an ELISA kit (R&D Systems Inc., Minneapolis, MN, USA). HSP70 was determined using a Stress Press ELISA Kit (Stress press®, USA).

### Western blot analysis

Cytoplasmic and nuclear proteins were extracted from frozen lung tissues with the Nuclear/Cytosol Extraction kit (BioVision, Inc., Mountain View, CA,USA) according to the manufacturer’s instructions. The protein concentrations were determined using the BCA protein assay kit (Pierce). Equal amounts of lung homogenates (30 μg/lane) were fractionated on 10% sodium dodecyl sulfate polyacrylamide gels and transferred to polyvinylidene fluoride membranes (Hybond; Amersham Biosciences, USA). The membranes were blocked by incubation in phosphate-buffered saline (PBS) containing 0.1%Tween 20 (Sigma-Aldrich, USA) and 5% nonfat milk for 1 h at room temperature. The blots were then incubated with rabbit anti-iNOS antibodies (EMD Millipore,USA), rabbit anti-NF-κB p65 (Cell Signaling Technology, USA), anti-phosphorylated-NF-κB p65 (Cell Signaling Technology, USA), anti-inhibitor of NF-κB (IκB)-α (Cell Signaling Technology, USA), anti-phosphorylated-IκB kinase (IKK)-α/β polyclonal antibodies (Cell Signaling Technology, USA), anti-IKKβ polyclonal antibodies (Cell Signaling Technology, USA), and an anti-HSP70 antibody (Abcam^®^, UK) for 1 h at room temperature. The blots were subsequently washed in PBST once for 20 minutes and twice for 10 minutes. After washing, the blots were incubated with anti-rabbit immunoglobulin IgG (1:20000) or anti-mouse IgG (1:50000) for 1 h at room temperature and then they washed in PBST once for 20 min and twice for 10 min. Protein bands were visualized using enhanced chemiluminescence reagents and by exposing the blots to X-ray film. The blots were then stripped and incubated with an anti-PCNA antibody (for nuclear proteins, diluted 1:10000; Cell Signaling Technology, USA) or an anti-β-actin antibody (for cytoplasmic proteins, diluted 1:10000; Sigma-Aldrich, USA) to ensure equal loading. The ratios of the band intensities were calculated.

### Experimental protocols

SD rats were continuously fed a 0.75% adenine diet to induce chronic kidney disease. After adenine feeding for two, four, or six weeks, blood urea, nitrogen, and creatinine levels were measured and renal pathology was assessed to confirm the development of adenine-induced CKD.

The rats were randomly assigned to groups constituting control, ischemia-reperfusion (IR) only, or IR with different durations of the 0.75% adenine diet (two, four, or six weeks). The isolated lungs were allowed to equilibrate for 20 min before analysis. The baseline PAP, PVP, weight change, and the initial *K*_f_ for 7 min were then measured. All parameters were equilibrated to baseline for 10 min after measurement. In the control group, the lungs were ventilated with humidified air containing 5% CO_2_ and were perfused with perfusate alone for 120 min. In the IR group, the lungs were ventilated with humidified air containing 5% CO_2_. After all parameters returned to the baseline values, the lungs were subjected to 30 min of ischemia by stopping ventilation and perfusion. Perfusion and ventilation were resumed, and the measurement of *K*_f_ was repeated 90 min later.

### Statistical analysis

The data are expressed as the mean ± standard deviation. Significant differences between group means were determined with a one-way repeated measures ANOVA, followed by a Scheffe's comparison post-hoc test. Comparisons within each group for a given parameter were performed using paired Student’s *t-*tests. Significance was considered to be present for values of *p* < 0.05.

## Results

### An experimental model of chronic kidney disease was verified to be induced by the adenine diet

**Fig 1:**
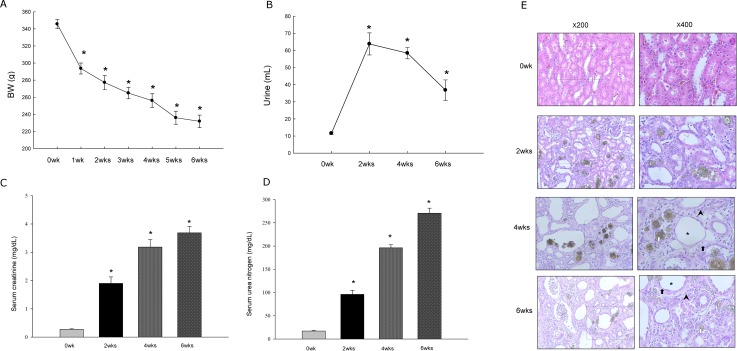
Adenine diet induces chronic kidney disease in rats. (A-E) Rats were fed an adenine diet for 6 weeks. (A) Time course of changes in body weight after feeding adenine diet. (B) Time course of changes in urinary output after feeding adenine diet.(C) Comparison of the blood urea nitrogen (BUN) in rats fed an adenine diet. (D) Serum creatinine levels in rats fed an adenine diet. (E) Representative photographs (×200 and ×400) of light microscopy showing sections of renal tissue from rats treated with a normal or adenine diet after hematoxylin and eosin staining. Renal histology showed deposition of crystalline structures in a tubular lumen (white arrow), dilated tubules (asterisk), diffuse polymorphic neutrophil infiltration (black arrowhead), tubular necrosis, tubular atrophy and interstitial fibrosis (black arrow). The data are expressed as the means ± SD. *Significantly different from the 0 week group (*p*<0.05). The time point of 0 week means baseline of rats without adenine diet

Consistent with previous observations reported by Yokozawa et al. [21], the mean body weight of the rats began to gradually decrease after one weeks of adenine challenge (Fig 1A). In the adenine-fed rats, the amount of urine produced increased significantly in response to the adenine challenge but decreased between the 2, 4 and 6 week cohorts, indicating that the rat kidneys attempted to respond to the challenge while gradually failing (Fig 1B). This hypothesis was confirmed when, compared with the 0 week (basline) group, there was a time-dependent increase in the serum urea nitrogen and creatinine levels in rats fed the adenine diet (Fig 1C and 1D). The histopathology of the kidney tissues showed prominent deposition of crystalline structures in the tubular lumens, diffuse tubular injury with neutrophil infiltration, tubular necrosis, tubular atrophy, interstitial inflammatory cell infiltration, and interstitial fibrosis in the adenine-fed rats (Fig 1E). Considering weight loss, reduced urine production, increased serum urea and pathological observations, these results confirmed that we successfully induced experimental CKD in SD rats by challenging them with a 0.75% adenine diet.

### Chronic kidney disease attenuates IR-induced acute lung injury

**Fig 2:**
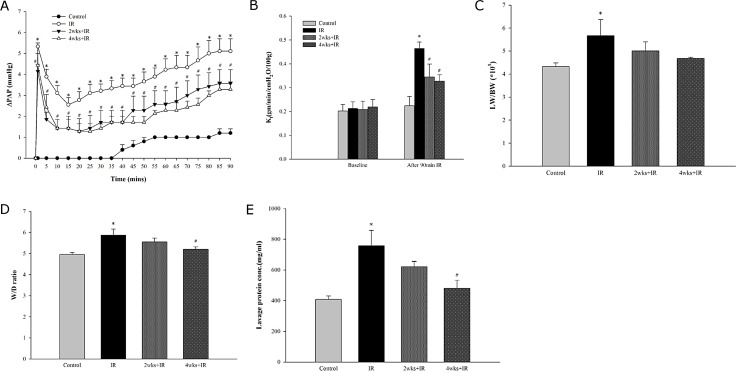
Chronic kidney disease attenuates the pulmonary edema induced by IR. The changes in (A) PAP, (B) *K*_f,_ (C) LW/BW ratio, (D) W/D weight ratio, and (E) protein concentration in BALF during reperfusion. The data are expressed as the means ± SD. *Significantly different from the control (P < 0.05); *#* Significantly different from the IR group (P < 0.05).

In the IR group, PAP increased after ischemia and reperfusion and then dropped to a trough at 15 min post-reperfusion. At 90 min post-reperfusion, the PAP was still significantly higher than in the control group. Compared with the LIRI rats fed a normal diet, the rats fed an adenine diet exhibited a significantly attenuated increase in IR-induced PAP (Fig 2A).

As shown in Fig 2B, we found that *K*_f_ after reperfusion was 1.9 times higher than baseline in the IR group (P<0.05), but it did not change significantly over a 120-min interval of perfusion in the control group. The increase in *K*_f_ in response to IR was significantly attenuated by in the 2 and 4 week adenine challenged rat cohorts (P<0.05).

IR injury caused a significant increase in the LW/BW ratios in all rat cohorts compared with the sham-operated control group. Compared with the IR group fed a normal diet, the increase in the lung weight was lower in rats fed the adenine diet for 2 or 4 weeks; however, the difference did not reach statistical significance (Fig 2C). Similarly, IR injury significantly induced an increase in the W/D lung weight ratios in LIRI rats compared with the sham-operated control group. The 4-week adenine-challenged LIRI rats exhibited a significant decrease in their W/D ratios compared with the IR group (Fig 2D). Moreover, the protein concentration in the BALF was significantly higher in the IR group than in the control group fed a normal diet. The 4 week adenine-challenged LIRI exhibited a significantly ameliorated increase in the BALF protein concentration (Fig 2E). These results indicate that the high vascular permeability and pulmonary edema induced by LIRI were attenuated in rats with adenine-induced CKD.

### Chronic kidney disease attenuates the histopathological lung injury induced by IR

**Fig 3:**
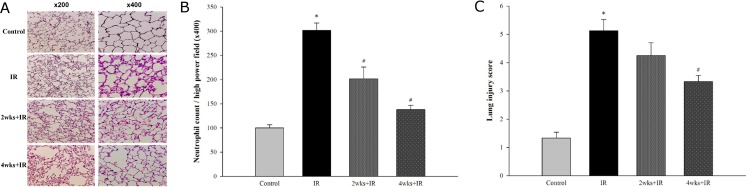
Chronic kidney disease improves the histopathological changes of the lung tissue induced by IR. The histological appearance of lung tissue samples from rats with IR-induced lung injury. (A) Micrographs of lung tissue (×200 and ×400 magnification), (B) Numbers of neutrophils per high power field (400X magnification), and (C) lung injury scores. The data are expressed as the means ± SD. *Significantly different from the control (*P* < 0.05); *#* Significantly different from the IR group (*P* < 0.05).

Compared with control rats, all cohorts of LIRI rats exhibited prominent thickening of the inter-alveolar septum and increased inflammatory cell infiltration (Fig 3A). A semi-quantitative assessment of lung inflammation and injury was performed by measuring the numbers of neutrophils (Fig 3B) and the lung injury scores (Fig 3C), which were significantly higher in the IR group than in the control group (P < 0.05). The adenine challenge resulted in a significant improvement in the histopathological changes in the lungs in the LIRI rats (Fig 3A) and a significant reduction in neutrophil infiltration (Fig 3B) and lung injury scores (Fig 3C).

### Chronic kidney disease improves the IHC changes of the lung tissue induced by IR

**Fig 4:**
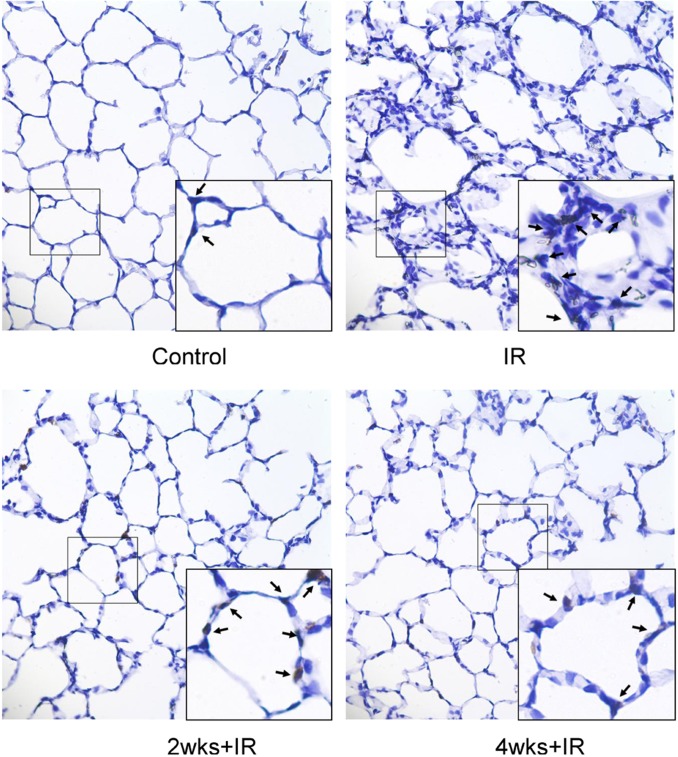
Chronic kidney disease improves the IHC changes of the lung tissue induced by IR. The MPO IHC staining of lung tissue samples from rats with IR-induced lung injury. The MPO IHC staining reveals infiltration of neutrophils into lung tissue (black arrow).The figure is presented at a magnification of X400 (insert, X1000).

The MPO IHC staining revealed that the number of neutrophils infiltration in the lung was higher in the IR group than in the control group whereas neutrophil infiltration was decreased in the 2 and 4 week adenine-challenged LIRI rats (Fig 4).

### Chronic kidney disease suppresses the expression of chemokine CXCL1 and neutrophil recruitment

**Fig 5:**
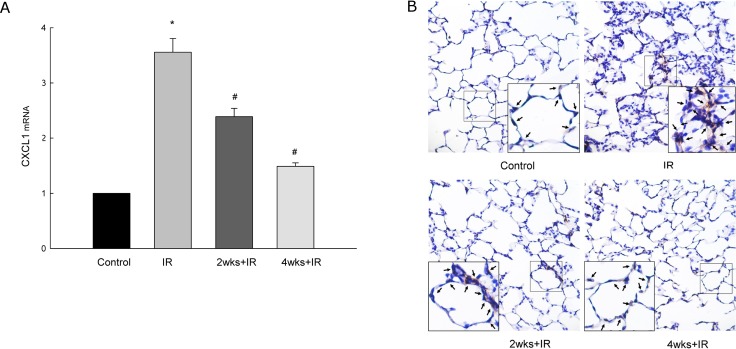
Chronic kidney disease suppresses the expression of chemokine CXCL1 and neutrophil recruitment. (A) the expression of CXCL1 mRNA in the lungs from rats with IR-induced lung injury. (B) CXCL1 IHC staining of lung tissue samples from rats with IR-induced lung injury. The CXCL1 IHC staining reveals neutrophils recruitment into lung tissue (black arrow).The data are expressed as the means ± SD. *Significantly different from the control (*P* < 0.05); *#* Significantly different from the IR group (*P* < 0.05).The figure is presented at a magnification of X400 (insert, X1000).

IR could induce the expression of CXCL1 mRNA in the lung. The expression of chemokine CXCL1 mRNA in the lung was suppressed in the 2 and 4 week adenine-challenged LIRI rats (Fig 5A). Furthermore, The CXCL1 IHC staining revealed that the number of neutrophils recruitment in the lung was higher in the IR group than in the control group where the number of neutrophils recruitment was decreased in the 2 and 4 week adenine-challenged IRLI rats (Fig 5B).

### Chronic kidney disease attenuates pulmonary lipid peroxidation induced by IR injury

**Fig 6:**
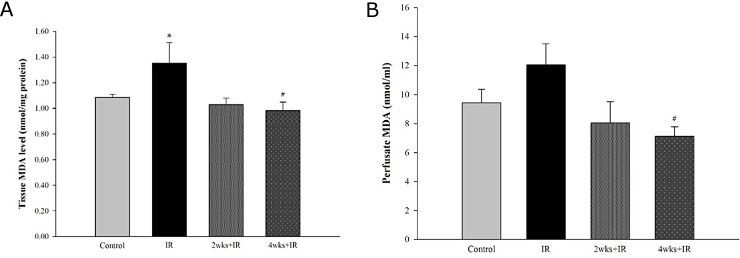
Effects of chronic kidney disease on IR-induced lung injury. The changes in (A) lung tissue MDA level and (B) perfusate MDA level. The data are expressed as the means ± SD. *Significantly different from the control (*P* < 0.05); *#* Significantly different from the IR group (*P* < 0.05).

IR group lung tissue MDA levels were significantly higher (Fig 6A), and perfusate MDA levels were somewhat higher compared with the control group (Fig 6B). The 4 week adenine-challenged rats’ increases in both tissue and perfusate MDA levels induced by IR injury were significantly abated (Fig 6A and 6B).

### Chronic kidney disease attenuates Inducible nitric oxide synthase (iNOS) induced by IR injury

**Fig 7:**
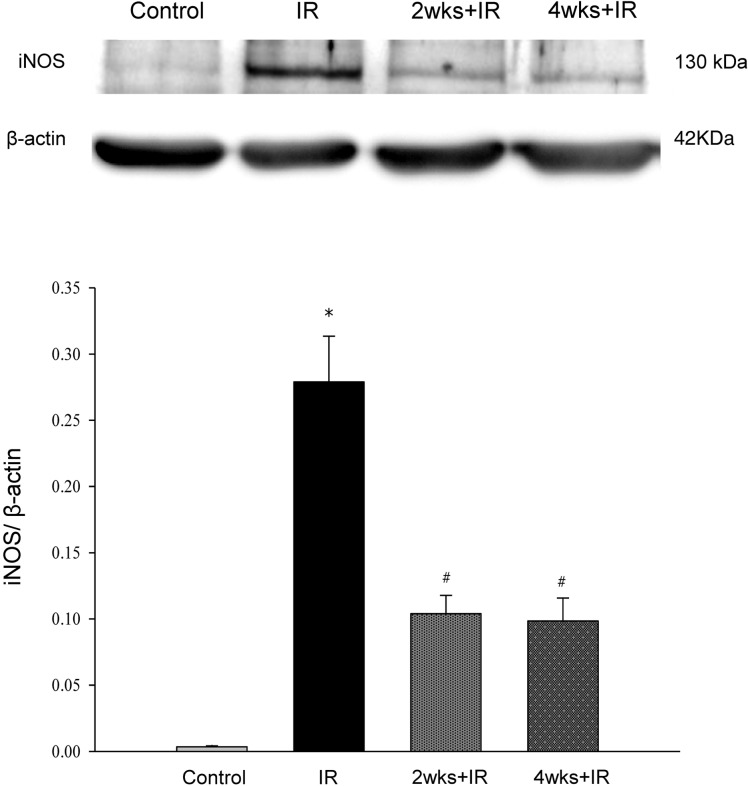
Effects of chronic kidney disease on inducible nitric oxide synthase induced by IR injury. The in iNOS level in the lung tissue from rats with IR-induced lung injury determined by western blotting. The data are expressed as the means ± SD. *Significantly different from the control (*P* < 0.05); *#* Significantly different from the IR group (*P* < 0.05).

In IR group, iNOS level of the lung was significantly higher compared with the control group. The iNOS level induced by IR injury was attenuated in the 2 and 4 week adenine-challenged rats (Fig 7).

### Chronic kidney disease suppresses the production of pro- and anti-inflammatory cytokines induced by IR injury

**Fig 8:**
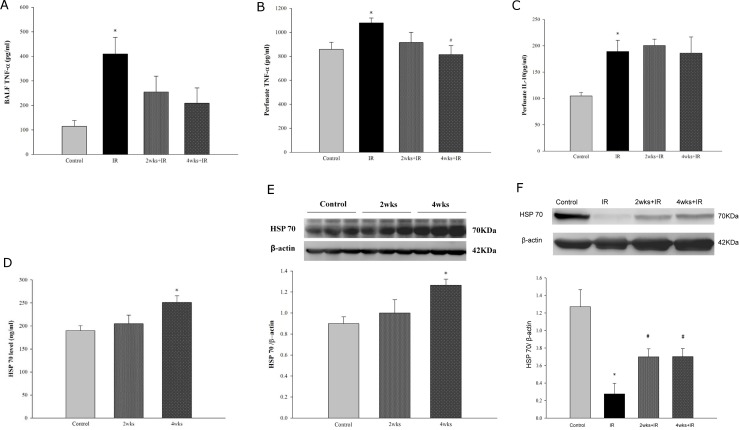
Effects of HSP70 on the protective effects of CKD against IR lung injury. (A) TNF-α level in the BAL; (B) TNF-α level in the perfusate; (C) IL-10 level in the perfusate; (D) HSP70 level in the lung tissue; (E) cytoplasmic HSP70 level in the lung tissue determined by western blotting; and (F) HSP70 level in the lung tissue determined by western blotting in IR induced lung injury. The data are expressed as the means ± SD. *Significantly different from the control (*P* < 0.05); *#* Significantly different from the IR group (*P* < 0.05).

As shown in Fig 8, we found that the TNF-α levels in the BALF (Fig 8A), perfusate (Fig 8B) and the IL-10 level in the perfusate (Fig 8C) were significantly increased in the IR group compared with those in the control group. The high levels of TNF-α in the perfusate were significantly suppressed in the 4 week adenine challenged LIRI rats (Fig 8B). Conversely, the CKD induced by the adenine challenge did not further alter IL-10 production compared with rats fed a normal diet. The lung tissue and cytoplasmic HSP70 levels determined by western blotting (Fig 8D and 8E) were significantly elevated in the 4 week adenine-challenged compared with the rats fed a normal diet. The HSP70 level in lung tissue (Fig 8F) determined by Western blotting was substantially decreased in the IR group versus the control group. However, the HSP70 level in lung tissue was significantly elevated in the 2 and 4 week adenine-challenged rats.

### Chronic kidney disease ameliorates the LIRI by inhibiting NF-κB signaling pathway

**Fig 9:**
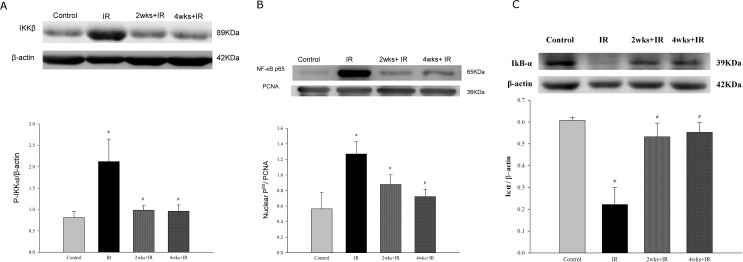
Effects of chronic kidney disease on NF-κB activation. **(A)**Cytoplasmic levels of phosphorylated IKK; (B) nuclear levels of total NF-κB p65; and (C) IκB-α level. The data are expressed as the means ± SD. *Significantly different from the control (*P* < 0.05); *#* Significantly different from the IR group (*P* < 0.05).

The cytoplasmic level of phosphorylated IKK and the nuclear level of NF-κB p65 were significantly increased following LIRI (Fig 9A and 9B), whereas the level of IκBα (Fig 9C) was significantly suppressed. Adenine-induced CKD markedly attenuated the decrease in IκBα expression and the increase in cytoplasmic phosphorylated IKK and nuclear NF-κB p65 expression in LIRI rats (Fig 9A–9C).

## Discussion

In the present study, we used an isolated lung *in situ* rat model to demonstrate that, as anticipated, acute lung injury induced by IR significantly increased the lung edema, PAP, neutrophil infiltration, iNOS level, inflammatory cytokine production, IκB-α degradation, nuclear translocation of NF-κB, and tissue injury. The IR-induced lung injury was significantly attenuated in animals with adenine-induced CKD. These data indicate that CKD may play a role in suppressing the inflammatory response and in attenuating the high-permeability pulmonary edema induced by IR injury.

Activation and migration of neutrophils into the lungs is one of the key events in ALI. Chemokine CXCL1 plays a crucial role in host immune response by recruiting and activating neutrophils for microbial killing at the tissue site. Several lines of evidence have shown that the chemokine CXCL1 plays a beneficial role by trafficking and activating neutrophils in response to infections, but is also implicated in exacerbating injury in lung tissue [22,23]. However, renal failure, and particularly chronic renal failure with the need for hemodialysis, also can alter neutrophil function. Furthermore, neutrophils from patients with chronic renal failure have been shown to display reduced phagocytic ability, and impaired chemotactic ability [24]. Indeed, our study indicates that CKD decreased neutrophil infiltration and suppressed the expression of chemokine CXCL1 induced by IR.

NF-κB is essential for activating the transcription of a pro-inflammatory cytokines and chemokine cascade that induces early inflammatory responses. The activation of NF-κB has been shown to be involved in the onset of pulmonary inflammation and to participate in the pathogenesis of LIRI [25]. In an experimental rat model of lung transplantation, Ishiyama *et al* demonstrated that inhibiting nuclear factor κB activation by gene transfer of IκB in a super-repressor form (IκBSR) improved the transplanted lung graft oxygenation and pulmonary edema and reduced the neutrophil sequestration and apoptotic cell death [26]. Similarly to previous studies, we found that LIRI significantly increased the pulmonary NF-κB p65 nuclear translocation, IKK phosphorylation and degradation of IκBα, while adenine-induced CKD markedly attenuated the activation of the NF-κB system in lung tissue. Furthermore, a previous report demonstrated that enhanced HSP70 expression alleviated the activation of NF-κB in sepsis and IR-induced lung injury in rats [27]. Collectively, these data suggest that CKD may ameliorate LIRI by inhibiting NF-κB signaling.

There is a growing belief that excessive nitric oxide (NO) production by iNOS plays an important role in the induction of lung injury in patients with adult respiratory distress syndrome (ARDS). Kristof and colleagues showed that mice deficient in iNOS gene are more resistant to LPS-induced acute lung injury than are wild-type mice [28]. On the other hand, the induction of iNOS may play a harmful role by directly inducing tissue damage and through the formation of peroxynitrite [29,30]. In addition, inhibitors of NOS activity have also been reported to attenuate lung injury and reduce the formation of peroxynitrite [31,32]. A critical role for the transcription factor NF-κB has been demonstrated in the transcriptional regulation of the murine and human iNOS gene induced by LPS and cytokines in cultured cells [33–36]. Liu and colleagues reported that LPS activated NF-κB in vivo, which, in turn, induced transcription of the iNOS gene and expression of the iNOS protein in a rat model of septic shock. They suggested that targeting NF-κB might be a effective strategy for the treatment of septic shock, because inhibition of NF-κB activation selectively prevented the increase in iNOS activity and iNOS-mediated NO production [37]. These findings may explain our present results that CKD attenuates iNOS related lung injury induced by IR.

The molecular mechanisms underlying the development of LIRI involve complex interactions that include the activation of leukocytes and the coagulation cascade, the production of pro-inflammatory cytokines, and the generation of reactive oxygen species. In addition to the induction of pro-inflammatory cytokines, previous studies have shown that LIRI also stimulates the production of anti-inflammatory mediators, such as IL-10 [38], which is a pluripotent cytokine that plays a pivotal role in the regulation of immune and inflammatory responses. IL-10 has been shown to alleviate the inflammatory response in various models of LIRI [39]. Although our results clearly demonstrated that LIRI was associated with an elevation of the IL-10 levels, adenine-induced CKD did not further enhance the IL-10 production compared to LIRI rats fed a normal diet. It has been shown that the kidney is the main organ responsible for the catabolism and excretion of cytokines, but the catabolism of IL-10 is difficult to predict in CKD kidneys. Furthermore, it is suggested that CKD may suppress the IL-10 mRNA expression in whole blood cells in patients with advanced CKD (GFR < 15 ml/min per 1.73 m^2^) [40]. Taken together, these findings and the extant literature suggest that IL-10 does not play a major role in modulating the course of LIRI in the rats with adenine-induced CKD.

The expression of the phylogenetically-conserved heat shock proteins (HSPs) in response to noxious stimuli is part of an endogenous system that modulates the systemic inflammatory responses [41]. Among these HSPs, HSP70 is highly inducible and has been demonstrated to play an essential role in normal cell processes and in the response to noxious stimuli [42]. In a rat model of cecal ligation and puncture to induce sepsis, glutamine ameliorated the lung injury and mortality after sepsis by enhancing lung heat shock factor-1 phosphorylation and HSP70 expression [43]. Similarly, the administration of an adenovirus expressing HSP70 (AdHSP) prevented or ameliorated lung injury in a rat model with sepsis-induced ARDS [44]. In this study, we found that the expression of HSP70 significantly decreased in lung tissue in LIRI rats, which was reversed by adenine-induced CKD, suggesting that HSP70 may play a role in the protective effects of CKD against LIRI. It has also been demonstrated that enhanced the expression of HSP70 attenuated the activation of NF-κB in rats with sepsis and IR-induced lung injury [27]. Our present results show that the HSP70 levels of lung tissue homogenates and its protein abundance determined by western blotting were gradually increased after challenging rats with an adenine diet. Taken together, these findings suggest that CKD may attenuate LIRI through the induction of HSP70 expression.

In summary, we demonstrated that CKD attenuated LIRI by improving lung edema and suppressing the production of inflammatory cytokines. Modulation of the NF-κB pathway, with reduced IκB-α degradation and limited NF-κB nuclear translocation, may underlie the protective effects of CKD against LIRI. Thus, whether patients with CKD can be potential donors for lung transplant warrants further investigation.

## Supporting information

S1 Fig Adenine diet induces chronic kidney disease with hyperkalemia in rats. The blood concentration of potassium (K^+^) was significantly higher in the adenine diet group than in the baseline group.*Significantly different from the 0 week group (*p*<0.05). The time point of 0 week means baseline of rats without adenine diet.
(TIF)
