# Supplementary figures and images for: Experimental chronic kidney disease attenuates ischemia-reperfusion injury in an ex vivo rat lung model
Source: PLoS One. 2017 Mar 14;12(3):e0171736. doi: 10.1371/journal.pone.0171736 (PMC5349449; doi:10.1371/journal.pone.0171736)

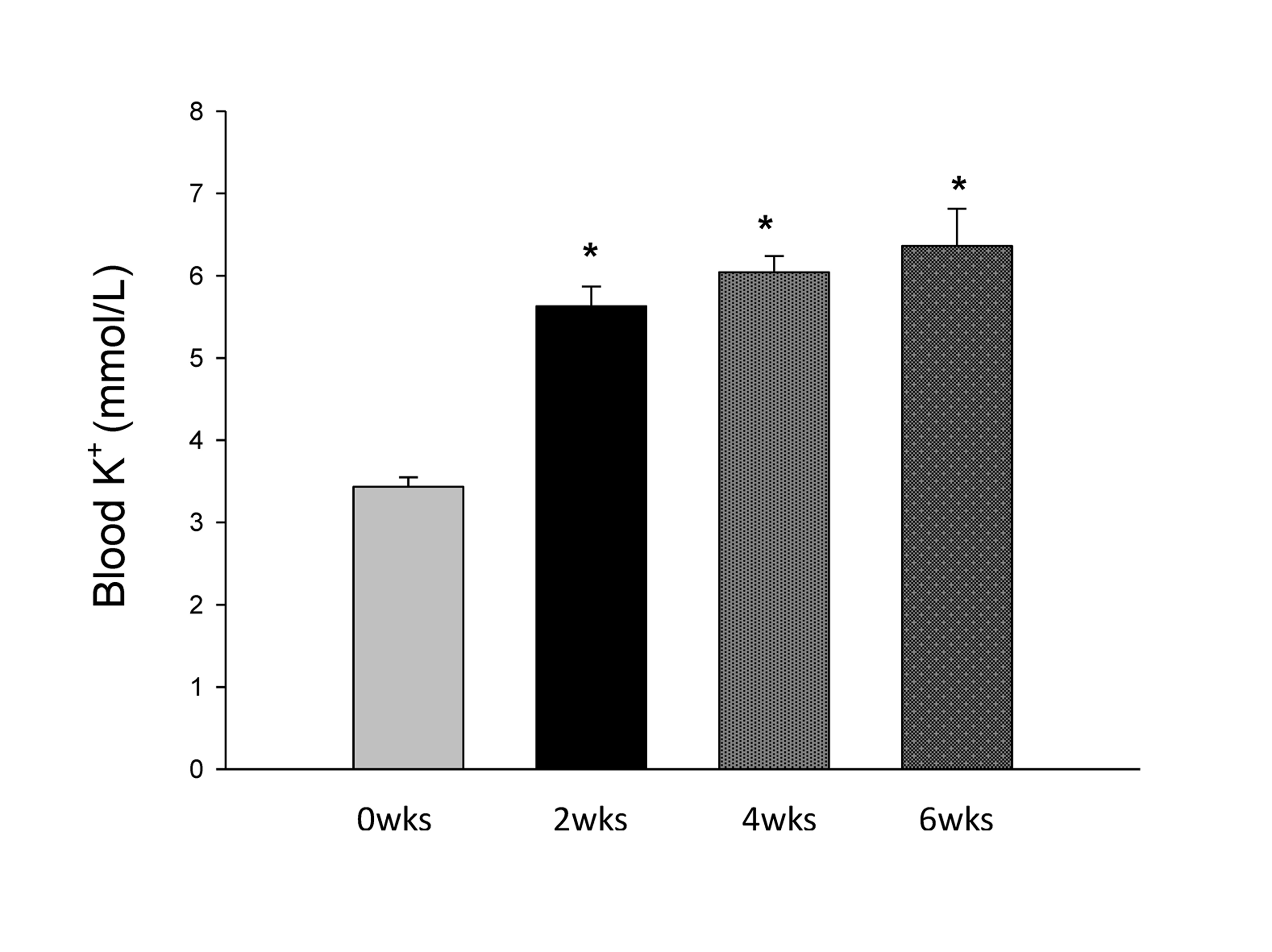

Supplement: S1 Fig — The blood concentration of potassium (K+) was significantly higher in the adenine diet group than in the baseline group.*Significantly different from the 0 week group (p<0.05). The time point of 0 week means baseline of rats without adenine diet. (TIF) [file pone.0171736.s001.tif]
